# Refined Locally Linear Transform-Based Spectral-Domain Gradient Sparsity and Its Applications in Spectral CT Reconstruction

**DOI:** 10.1109/access.2021.3071492

**Published:** 2021-04-07

**Authors:** QIAN WANG, MORTEZA SALEHJAHROMI, HENGYONG YU

**Affiliations:** Department of Electrical and Computer Engineering, University of Massachusetts at Lowell, Lowell, MA 01854, USA

**Keywords:** Refined locally linear transform, structural similarity, sparsity construction, spectral-dimension gradient sparsity, constrained optimization, iterative reconstruction, spectral CT, material decomposition

## Abstract

Spectral computed tomography (CT) is extension of the conventional single spectral CT (SSCT) along the energy dimension, which achieves superior energy resolution and material distinguishability. However, for the state-of-the-art photon counting detector (PCD) based spectral CT, because the emitted photons with a fixed total number for each X-ray beam are divided into several energy bins, the noise level is increased in each reconstructed channel image, and it further leads to an inaccurate material decomposition. To improve the reconstructed image quality and decomposition accuracy, in this work, we first employ a refined locally linear transform to convert the structural similarity among two-dimensional (2D) spectral CT images to a spectral-dimension gradient sparsity. By combining the gradient sparsity in the spatial domain, a global three-dimensional (3D) gradient sparsity is constructed, then measured with *L*_1_-, *L*_0_- and trace-norm, respectively. For each sparsity measurement, we propose the corresponding optimization model, develop the iterative algorithm, and verify the effectiveness and superiority with real datasets.

## I. INTRODUCTION

X-ray computed tomography (CT), as a nondestructive inspection technique, has been widely used in many fields, such as medicine, industry, biology, exploration, security, and so on [1]–[3]. Although the specific equipment design, scan protocol, and utilization purpose are greatly diverse, the fundamental image-forming principle is consistently based on the Beer–Lambert law, which describes the relation between X-ray and the object as an exponential attenuation model,

(1) $$I\left(E\right)=I_{0}\left(E\right)\text{exp}\left(-P\left(\mu \left(E,x\right)\right)\right),$$

where *µ*(*E, x*) is the linear attenuation coefficient for energy *E* at the position *x* [4], P(⋅) a line integral operator (the ray transform and especially the Radon transform for 2D cases [5]), *I*_0_(*E*) the emitted photons, and *I*(*E*) the collected photons. Actually, the validity of (1) is confined to a set of ideal imaging conditions, such as the X-ray energy is monochromatic, there is no scattering in the imaging process, and so on. However, in practical applications, the energy *E* obeys an emitted spectral distribution called *S*(*E*), i.e., polychromatic. Thus, (1) should be changed to

$$I=\int_{E}S\left(E\right)\text{exp}\left(-P\left(\mu \left(E,x\right)\right)\right)\text{d}E.$$

After the logarithmic transformation, we can obtain the commonly known projection as follow,

(2) $$P=\text{ln}\left[\frac{\int_{E}S\left(E\right)\text{d}E}{\int_{E}S\left(E\right)\text{exp}\left(-P\left(\mu \left(E,x\right)\right)\right)\text{d}E}\right].$$

The problem of CT reconstruction is to determine an approximate distribution of *µ* from a set of (2).

Over the last 40 years, 5 major innovations were made in the development of CT technology, which contribute to improve the scan efficiency and practicability, i.e., spatial-domain modification, but never touch the spectral scope. In other words, these generations just develop different patterns to construct P(⋅) mappings. Recently, spectral CT is proposed by extending the conventional single-spectral computed tomography (SSCT) along the energy dimension [6]. And the state-of-the-art implementation technique is employing a photon-counting detector (PCD), a kind of energy-selective detector [7], [8], which can divide the X-ray photons into different energy channels with appropriate post-processing steps and then obtain multiple energy-dependent projection sets [9]. Thus, compared with the SSCT, spectral CT is superior in energy resolution and material distinguishability. It has great potential in both medical and industrial applications [10]. Mathematically, spectral CT can be described by introducing a window function to (2), i.e.,

(3) $$P_{c}=\text{ln}\left[\frac{\int_{E}S\left(E\right)W_{c}\left(E\right)\text{d}E}{\int_{E}S\left(E\right)W_{c}\left(E\right)\text{exp}\left(-P\left(\mu \left(E,x\right)\right)\right)\text{d}E}\right].$$

Here

$$W_{c}\left(E\right)=\left\{ \begin{array}{l} 1,\;E\in \omega_{c},\\ 0,\;\text{otherwise}, \end{array}\right.$$

is a window function of the channel *c*, 1 ≤ *c ≤ C*, *ω*_*c*_ indicates the energy interval, and *C* is the channel number.

By denoting the inverse operator of P(⋅) as $P^{-1}\left(\cdot \right)$, we can obtain the channel-wise image as follow,

$$F_{c}\left(x\right)\;\;=P^{-1}\left(P_{c}\right)\;\;=P^{-1}\left\{-\text{ln}\left[\frac{\int_{E}S\left(E\right)W_{c}\left(E\right)\text{exp}\left(-P\left(\mu \left(E,x\right)\right)\right)\text{d}E}{\int_{E}S\left(E\right)W_{c}\left(E\right)\text{d}E}\right]\right\}.$$

Denote the size of *F*_*c*_(1 ≤ *c ≤ C*) as *M × N*. By stacking up all the channel images along the spectral dimension, we can obtain a volumetric spectral image *F* with the size of *M × N × C*. Same strategy can be used for channel-wise projection data to achieve the corresponding volumetric spectral projection *P*.

Apparently, (2) can be treated as a special case of (3) when the only energy window is extended to fully cover the whole spectrum. And (3) represents the local performance of (2) with a truncated spectrum. Meanwhile, it is obvious that the emitted photons for a channel *c* are just a fraction of the originally emitted photons in an X-ray beam. Thus, in practical applications, the decreased channel dose inevitably increases the noise level of the corresponding projection. Thus, the fundamental problem of CT, i.e., how to reconstruct high-quality images from noisy projections, will be more challenging for spectral CT. Moreover, it may further have adverse consequences on the decomposition accuracy and damage the native material distinguishability.

To overcome the ill-posedness in spectral CT reconstruction, prior knowledge needs to be greatly concerned and effectively incorporated. The features of spectral CT images can be classified into two categories, which lie in the spatial and the spectral domains, respectively. The spatial feature can be ascribed to a sparsity in the spatial domain itself [11], an appropriate transform domain [12], [13], or a high-dimensional space [14]–[16]. The spectral feature is a correlation among channel images, more specifically, a structural similarity [17]–[20]. Most existing methods for spectral CT employ different measurements to directly describe both or either the aforementioned features.

Different from directly employing the correlation among channel images, in our previous study [21], we developed a locally linear transform based gradient *L*_0_-norm minimization method for spectral CT reconstruction. As a natural continuation and deeper investigation, in this work, we innovatively refine the proposed locally linear transform with a Gaussian kernel to improve the edge preservation, when converting the spectrum-related structural similarity to a gradient sparsity. Then, we propose a general optimization framework with a global three-dimensional (3D) sparsity constraint. We also concretize the 3D constraint with gradient *L*_1_- and *L*_0_-norm, and spatial total-variation with spectral trace-norm (TVLR) [14], respectively. Moreover, we perform experiments to verify the effectiveness and superiority of the proposed methods comparing with the previous versions (2D *L*_1_- and *L*_0_-norm minimization and TVLR method).

The remainder of this paper is organized as follows. In section II, we briefly review the locally linear transform based 3D gradient *L*_0_-norm minimization method for spectral CT. In section III, we present the refining strategy for the locally linear transform, establish a general optimization framework with 3D sparsity constraint, and develop concritized optimization models with gradient *L*_1_- and *L*_0_-norm, and TVLR. We perform real experiments to verify the effectiveness of the proposed method in section IV. In last section, we conclude this work.

## II. THEORY

In this section, we review how to construct a 3D gradient sparsity by employing the locally linear transform, and how to incorporate this sparse constraint into an optimization model.

### A. LOCALLY LINEAR TRANSFORM BASED 3D GRADIENT SPARSE CONSTRAINT

Comparing all the reconstructed channel images, the structural similarity and quantitative difference are obvious. By employing locally linear transform, we convert this feature to a gradient sparsity along the spectral direction. Assuming the current target channel is *c*, we fix *F*_*c*_ as the filtered input image, choose *F*_*i*_ (1 ≤ *i* ≤ *C*) as a reference image, and obtain a filtered output $F_{i}^{c}$ satisfying

(4) $$F_{i}^{c}\left(x\right)=a_{i}^{c}\left(k\right)F_{i}\left(x\right)+b_{i}^{c}\left(k\right),\;\forall x\in \Omega_{2}\left(k\right),$$

where *x* and *k* indicate pixel positions, Ω_2_(*k*) represents an image patch with a center position *k*. $\left(a_{i}^{c}\left(k\right),b_{i}^{c}\left(k\right)\right)$ is a pair of constant coefficients for the patch Ω_2_(*k*), which are determined by a quadratic optimization model as follow [22],

(5) $$\underset{\left(a\left(k\right),b\left(k\right)\right)}{\text{min}}\sum_{x\in \Omega_{2}\left(k\right)}\left[{\left(a_{i}^{c}\left(k\right)F_{i}\left(x\right)+b_{i}^{c}\left(k\right))-F_{c}\left(x\right)\right)}^{2}+\epsilon {\left(a_{i}^{c}\left(k\right)\right)}^{2}\right].$$

Equation (5) also suggests $F_{i}^{c}$ can be viewed as a copy of *F*_*c*_. Considering each pixel *x* is covered by several patches (∀*x* ∈ Ω_2_(*k*)), we adopt an averaging strategy for $\left(a_{i}^{c}\left(k\right),b_{i}^{c}\left(k\right)\right)$, i.e., $\left({\bar{a}}_{i}^{c}\left(x\right),{\bar{b}}_{i}^{c}\left(x\right)\right)$ is a pair of averaged coefficients in all the patches covering the pixel *x*. Thus, (4) is converted to

(6) $$F_{i}^{c}\left(x\right)={\bar{a}}_{i}^{c}\left(x\right)F_{i}\left(x\right)+{\bar{b}}_{i}^{c}\left(x\right),\;\forall x\in \Omega_{2}.$$

By performing the patch-wise parameter average, the locally linear transform in (4) is converted to the point-point linear transform in (6).

When we employ all the channel images as references, we can repeatedly perform (5) to obtain the corresponding filtered outputs. By stacking them up along the spectral direction, we can get a volumetric image *F*^*c*^, of which the *i*-th channel image is $F_{i}^{c}\left(1\leq i\leq C\right)$. Same operation can be performed to ${\bar{a}}_{i}^{c}$ and ${\bar{b}}_{i}^{c}\left(1\leq i\leq C\right)$, and we can also obtain the volume-based ${\bar{a}}^{c}$ and ${\bar{b}}^{c}$. Thus, we further represent (6) in a volume version as follow,

$$F^{c}\left(x\right)={\bar{a}}^{c}\left(x\right)F\left(x\right)+{\bar{b}}^{c}\left(x\right),\;\forall x\in \Omega_{3}.$$

Here *Ω*_3_ indicates the 3D spatial domain. The filtering input volume is represented as *V*^*c*^, which is a simply duplicate extension of *F*_*c*_ in the spectral dimension.

### B. OPTIMIZATION MODEL AND ITERATIVE ALGORITHM

To measure the 3D gradient sparsity of *F*^*c*^ by *L*_0_-norm, we employ a counting function C(⋅) as follow,

$$C\left(F^{c}\right)=\#\left\{x\in \Omega_{3}|\left|\partial_{x}F^{c}\left(x\right)\right|+\left|\partial_{y}F^{c}\left(x\right)\right|+\left|\partial_{z}F^{c}\left(x\right)\right|\neq 0\right\}.$$

Incorporating the data constraint and considering the relationship between *F*^*c*^ and *F*, we propose the following optimization model,

(7) $$\underset{F^{c}}{\text{min}}\left\{{\left\|P\left(F\right)-P\right\|}_{L_{2}}^{2}+\text{λ}C\left(F^{c}\right)\right\},\;\text{s.t.}\;F^{c}\left(x\right)={\bar{a}}^{c}\left(x\right)F\left(x\right)+{\bar{b}}^{c}\left(x\right),$$

where *λ*>0 is a parameter to control the importance of the regularization term. By relaxing the constraint, Eq. (7) is converted to the following unconstrained model,

(8) $$\underset{F,F^{c}}{\text{min}}\{{\left\|P\left(F\right)-P\right\|}_{L_{2}}^{2}+\text{λ}C\left(F^{c}\right)\;\;+\tau \sum_{x\in \Omega_{3}}{\left[F^{c}\left(x\right)-\left({\bar{a}}^{c}\left(x\right)F\left(x\right)+{\bar{b}}^{c}\left(x\right)\right)\right]}^{2}\},$$

where *τ >* 0 is a parameter controlling the relaxation degree. Then, for each target channel *c* (1 ≤ *c ≤ C*), we split (8) to the following sub-problems,

(9a) $$\underset{F}{\text{min}}\left\{{\left\|P\left(F\right)-P\right\|}_{L_{2}}^{2}+\tau \sum_{x\in \Omega_{3}}{\left[F^{c}\left(x\right)-\left({\bar{a}}^{c}\left(x\right)F\left(x\right)+{\bar{b}}^{c}\left(x\right)\right)\right]}^{2}\right\},$$

(9b) $$\underset{F^{c}}{\text{min}}\left\{\text{λ}C\left(F^{c}\right)+\tau \sum_{x\in \Omega_{3}}{\left[F^{c}\left(x\right)-\left({\bar{a}}^{c}\left(x\right)F\left(x\right)+{\bar{b}}^{c}\left(x\right)\right)\right]}^{2}\right\}.$$

Equation (9a) is a quadratic optimization problem, which can be iteratively solved by using the POCS scheme [23]. Equation (9b) can be viewed as a 3D generalization of the 2D gradient *L*_0_-norm minimization, and the solution approach can be achieved by extending the 2D method in [24]. Finally, by averaging *F*^*c*^ along the spectral dimension, we can obtain the searched-for channel image. Furthermore, the decomposition method [25] can be employed to obtain material percentage images.

## III. METHOD

In this section, we first refine the construction of the 3D gradient sparsity, i.e., refined locally linear transform. Then, we propose a general optimization framework with 3D gradient sparsity, and concrete it with three different regularizers (gradient *L*_1_- and *L*_0_-norm, and TVLR).

### A. REFINED SPECTRAL-DOMAIN GRADIENT SPARSITY CONSTRUCTION METHOD

The correlations among channel images are conspicuous. For one fact, all the slices contain the same structures and textures, i.e., **structural similarity**. For the other fact, the gray value and contrast vary a lot, i.e., **quantitative diversity**. When employing the locally linear transform to establish the 3D gradient sparsity, we perform a patch-wise average operation for the coefficient pair $\left(a_{i}^{c}\left(k\right),b_{i}^{c}\left(k\right)\right)$, which meanwhile may cause edge deformation. To overcome this drawback, we introduce a Gaussian kernel to refine the transform, i.e., replacing the average weight with a radial basis function. Thus, we revise (6) as

(10) $$F_{i}^{c}\left(x\right)={\tilde{a}}_{i}^{c}\left(x\right)F_{i}\left(x\right)+{\tilde{b}}_{i}^{c}\left(x\right),\;\forall x\in \Omega_{2},$$

where the tilde symbol indicates a weighted average operation. Be giving the central pixel more weight, and the edge pixel less weight, the edge distortion can be effectively suppressed.

When the reference image traverses all the energy channels, we can stack up the corresponding filtering outputs along the spectral direction to form a volume *F*^*c*^, of which the *i*-th channel image is $F_{i}^{c}\left(1\leq i\leq C\right)$. Thus, we further represent (10) in a volume version as follow,

$$F^{c}\left(x\right)={\tilde{a}}^{c}\left(x\right)F\left(x\right)+{\tilde{b}}^{c}\left(x\right),\;\forall x\in \Omega_{3}.$$

The corresponding filtering input volume is represented as *V*^*c*^, which is a duplicate extension of *F*_*c*_ along the spectral dimension. It is emphasized while *F*_*c*_ represents a 2D channel image, *F*^*c*^ is the corresponding 3D extension along the spectral dimension. It is worth noting that the channel images of *F*^*c*^ successfully overcome the shortcoming of quantitative diversity, and well maintain the structural similarity at the same time. Thus, its 3D gradient volume is globally sparse, i.e., 2D spatial sparsity and 1D spectral sparsity.

### B. REFINED LOCALLY LINEAR TRANSFORM BASED GENERAL OPTIMIZATION FRAMEWORK

Considering the gradient sparsity of *F*^*c*^ (1 ≤ *c ≤ C*), we employ it as a constraint by performing a general measurement noted as Φ(⋅). Combining the data fidelity term, we propose the following optimization framework,

(11) $$\underset{F,F^{c}}{\text{min}}\left\{{\left\|P\left(F\right)-P\right\|}_{L_{2}}^{2}+{\text{λ}}_{c}\Phi \left(F^{c}\right)\right\},\;\text{s.t.}\;F^{c}\left(x\right)={\tilde{a}}^{c}\left(x\right)F\left(x\right)+{\tilde{b}}^{c}\left(x\right),1\leq c\leq C.$$

Here *F* is the spectral CT reconstruction volume by stacking up all the channel images *F*_*c*_ (1 ≤ *c ≤ C*) along the spectral dimension. *F*^*c*^ is a duplication volume of the searched-for *c*-th channel image along the spectral dimension. ${\tilde{a}}^{c}\left(x\right)$ and ${\tilde{b}}^{c}\left(x\right)$ are determined by the following quadratic optimization model,

$$\underset{\left(a^{c}\left(x\right),b^{c}\left(x\right)\right)}{\text{min}}\left\{\sum_{t\in \Omega_{2}\left(x\right)}\left[{\left(a^{c}\left(x\right)F\left(t\right)+b^{c}\left(x\right))-V^{c}\left(t\right)\right)}^{2}+\epsilon {\left(a^{c}\left(x\right)\right)}^{2}\right]\right\}.$$

Similar to II-B, (11) can be relaxed and splitted into two sub-problems. The corresponding pseudo-codes are summarized in Algorithm 1.

$$\begin{array}{l} \bar{\text{Algorithm}\;\;1\;\;\text{Algorithm of the Refined Locally Linear}\;\;\;\;\;\;\;\;\;\;\;\;\;\;\;\;\;\;\;\;\;\;\;\;\;\;\;\;\;\;\;\;\;\;\;\;\;\;\;\;\;\;\;\;\text{​}\;\;\;\;\;\;\;\;\;}\\ \underline{\text{Transform}\;\;\text{Based}\;\;\text{Optimization}\;\;\text{Framework }\;\;\;\;\;\;\;\;\;\;\;\;\;\;\;\;\;\;\;\;\;\;\;\;\;\;\;\;\;\;\;\;\;\;\;\;\;\;\;\;\;\;\;\;\;\;\;\;\;\;\;\;\;\;}\\ \;\;F^{\left(0\right)}=0;\\ \;\;\text{for}\;\text{end}\\ \;\;i=0\;\;\text{to}\;{\text{Iter}}_{max}-1\;\text{do}\\ \;\;\;\;\;\;\;\text{for}\;c=1\;\text{to}\;C\;\text{do}\\ \;\;\;\;\;\;\;\;\;\;\;\;\;\text{Calculate}{\left({\tilde{a}}^{\left(i\right)}\right)}^{c}\text{and}{\left({\tilde{b}}^{\left(i\right)}\right)}^{c}\\ \;\;\;\;\;\;\;\;\;\;\;\;\;F^{\left(*c\right)}=\text{arg}{\text{min}}_{F}\{{\left\|P\left(F\right)-P\right\|}_{L_{2}}^{2}+\tau \sum_{x\in \Omega_{3}}[{\left(F^{\left(i\right)}\right)}^{c}\left(x\right)-\\ \;\;\;\;\;\;\;\;\;\;\;\;\;\;{\left({\left({\tilde{a}}^{\left(i\right)}\right)}^{c}\left(x\right)F\left(x\right)+{\left({\tilde{b}}^{\left(i\right)}\right)}^{c}\left(x\right)\right)]}^{2}\}\\ \;\;\;\;\;\;\;\text{end}\;\;\text{for}\\ \;\;\;\;\;\;\;F^{\left(*\right)}=\frac{1}{C}\sum_{c=1}^{C}F^{\left(*c\right)}\\ \;\;\;\;\;\;\;\text{for}\;c=1\;\text{to}\;C\;\text{do}\\ \;\;\;\;\;\;\;\;\;\;\;\;\;\text{Calculate}{\left({\tilde{a}}^{\left(*\right)}\right)}^{c}\text{and}{\left({\tilde{b}}^{\left(*\right)}\right)}^{c}\\ \;\;\;\;\;\;\;\;\;\;\;\;{\left(F^{\left(*+1\right)}\right)}^{c}={\text{arg min}}_{F^{c}}\{\text{λ}\Phi (F^{c})+\tau \sum_{x\in \Omega_{3}}[F^{c}\left(x\right)-({\left({\tilde{a}}^{\left(*\right)}\right)}^{c}(x)F^{\left(*\right)}(x)+\\ \;\;\;\;\;\;\;\;\;\;\;\;\;{{\left({\tilde{b}}^{\left(*\right)}\right)}^{c}\left(x\right))]}^{2}\}\\ \;\;\;\;\;\;\;\;\;\;\;\;F_{c}^{i+1}=\frac{1}{C}\sum_{k=1}^{C}{\left(F^{\left(*+1\right)}\right)}_{k}^{c}\\ \;\;\;\;\;\;\;\text{end}\;\;\text{for}\\ \;\;\;\;\;\;\;i=i+1\\ \underline{\;\;\text{end}\;\;\text{for}\;\;\;\;\;\;\;\;\;\;\;\;\;\;\;\;\;\;\;\;\;\;\;\;\;\;\;\;\;\;\;\;\;\;\;\;\;\;\;\;\;\;\;\;\;\;\;\;\;\;\;\;\;\;\;\;\;\;\;\;\;\;\;\;\;\;\;\;\;\;\;\;\;\;\;\;\;\;\;\;\;\;\;\;\;\;\;\;\;\;\;\;\;\;\;\;\;\;\;\;\;\;\;\;\;\;\;\;\;\;\;\;\;\;\;\;\;\;\;\;\;\;\;\;\;\;\;} \end{array}$$

### C. CONCRETIZED 3D REGULARIZERS

Many measurement methods can be used to concretize the general regularizer Φ(⋅), such as *L*_1_-, *L*_0_-norm. However, the commonly employed version is 2D, which should be modified to a 3D extension to meet the sparsity feature in this problem.

- 3D gradient *L*_1_-norm:
  $$\Phi_{1}\left(F^{c}\right)=\sum_{x\in \Omega_{3}}\left|\partial_{x}F^{c}\left(x\right)\right|+\left|\partial_{y}F^{c}\left(x\right)\right|+\left|\partial_{z}F^{c}\left(x\right)\right|,$$
  where *Ω*_3_ is the spectral volume range.
- 3D gradient *L*_0_-norm:
  $$\Phi_{0}\left(F^{c}\right)=\#\left\{x\in \Omega_{3}|\left|\partial_{x}F^{c}\left(x\right)\right|+\left|\partial_{y}F^{c}\left(x\right)\right|\;\;+\left|\partial_{z}F^{c}\left(x\right)\right|\neq 0\right\},$$
  where #(⋅) counts the number of pixels satisfying $\left|\partial_{x}g\left(\cdot \right)\right|+\left|\partial_{y}g\left(\cdot \right)\right|+\left|\partial_{z}g\left(\cdot \right)\right|\neq 0$.

For the TVLR method [14], the trace-norm measurement is performed on a directly unfolded channel image according to the spectral domain. Theoretically, the trace-norm measurement describes a low-rank feature, which fits sparsity better than similarity. Thus, we modify the TVLR method by performing the trace-norm measurement on the unfolded *F*^*c*^ instead of *F*.

- Modified trace-norm:
  $$\Phi_{*}\left(F^{c}\right)={\left\|F_{\left(3\right)}^{c}\right\|}_{*}=\sum_{\gamma }\sigma_{\gamma }\left(F_{\left(3\right)}^{c}\right),$$
  where $\sigma_{\gamma }\left(F_{\left(3\right)}^{c}\right)$ is the *γ* -th largest singular value, $F_{\left(3\right)}^{c}={\text{unfold}}_{3}\left(F^{c}\right)\in R^{D_{3}\times D_{1}D_{2}}$, *D*_3_ is the spectral dimension, and *D*_1_ × *D*_2_ represents the spatial domain.

For each concretized Φ(⋅), we develop the corresponding optimization model and perform experiments to verify the effectiveness.

## IV. RESULTS

We perform two real experiments to verify the effectiveness of algorithm 1 concretized with the *L*_1_- and *L*_0_-norm and TVLR, respectively. We visually compare reconstructed channel images and decomposed material images among conventional filtered backprojection (FBP) method, TV-class methods, *L*_0_-class methods, and TVLR-class methods. The TV- and *L*_0_-class methods include 2D version, 3D version, locally linear transform (LLT-) based version and refined locally linear transform (ELLT-) based version. The TVLR-class methods include TVLR, locally linear transform (LLT-) based version and refined locally linear transform (ELLT-) based version. For all the aforementioned methods, we consistently fixed the iteration number to 20 for fair comparisons. We used an image-domain material decomposition method for all the experiments [26]. To quantitatively compare the decomposition accuracy, for each experiment, we calculated the mean value and standard deviation of the decomposed solid water for all the comparison methods.

The experiments are with a same X-ray source (YXLON 225 kV micro-focus tube) operated at a tube voltage of 140 kV and a tube current of 100 *µ*A. The detector is a 4-channel PILATUS3 PCDs by DECTRIS. The source-object distance is 35.27 cm and the source-detector distance is 43.58 cm. 720 views are collected by the detector consisting of 515 cells with 0.15 mm length. The reconstructed and decomposed results are with 512×512 pixels. For each pixel, the physical dimension is 0.122 mm × 0.122 mm.

**FIGURE 1.**
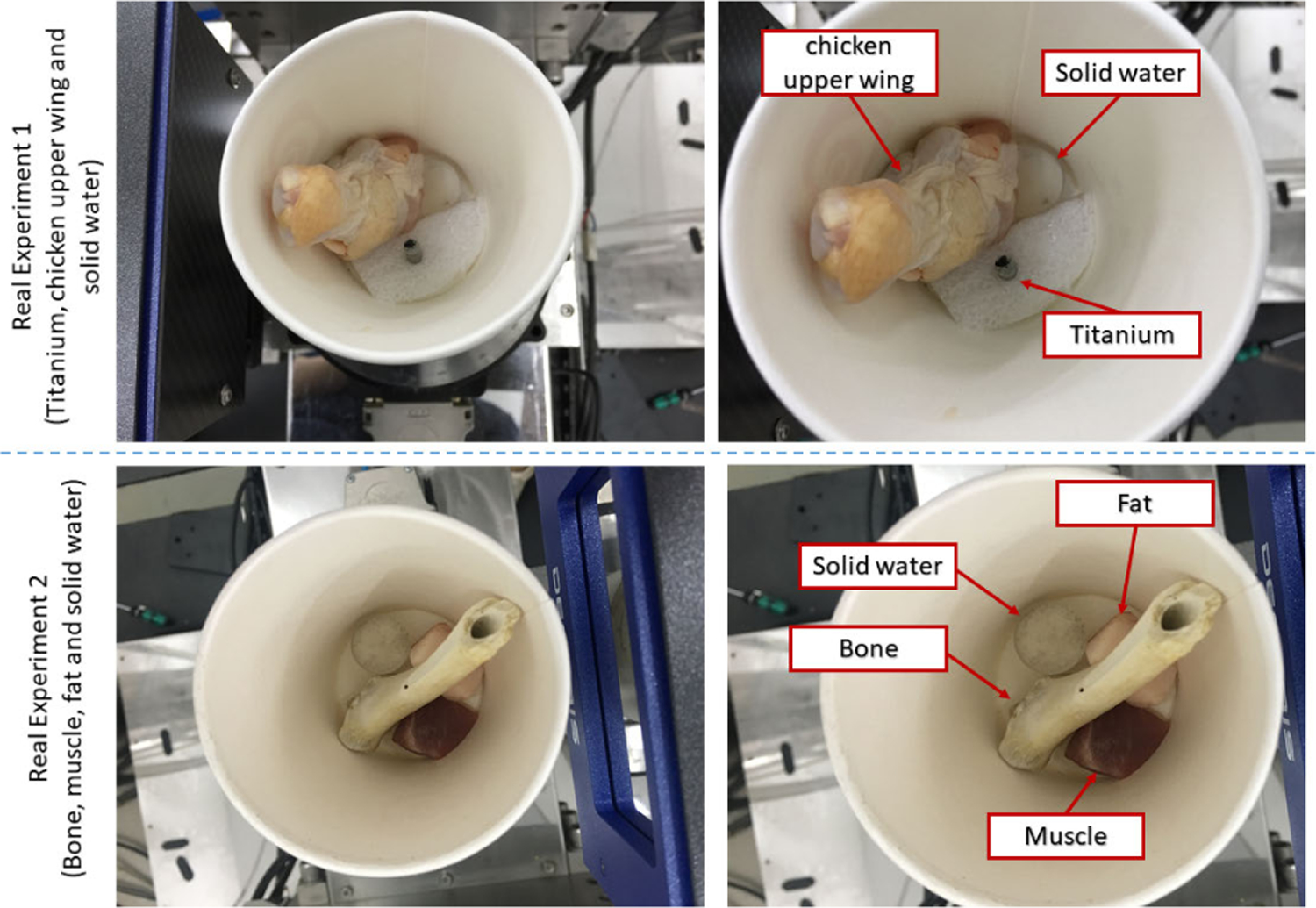
Scan settings for real experiments.

**FIGURE 2.**
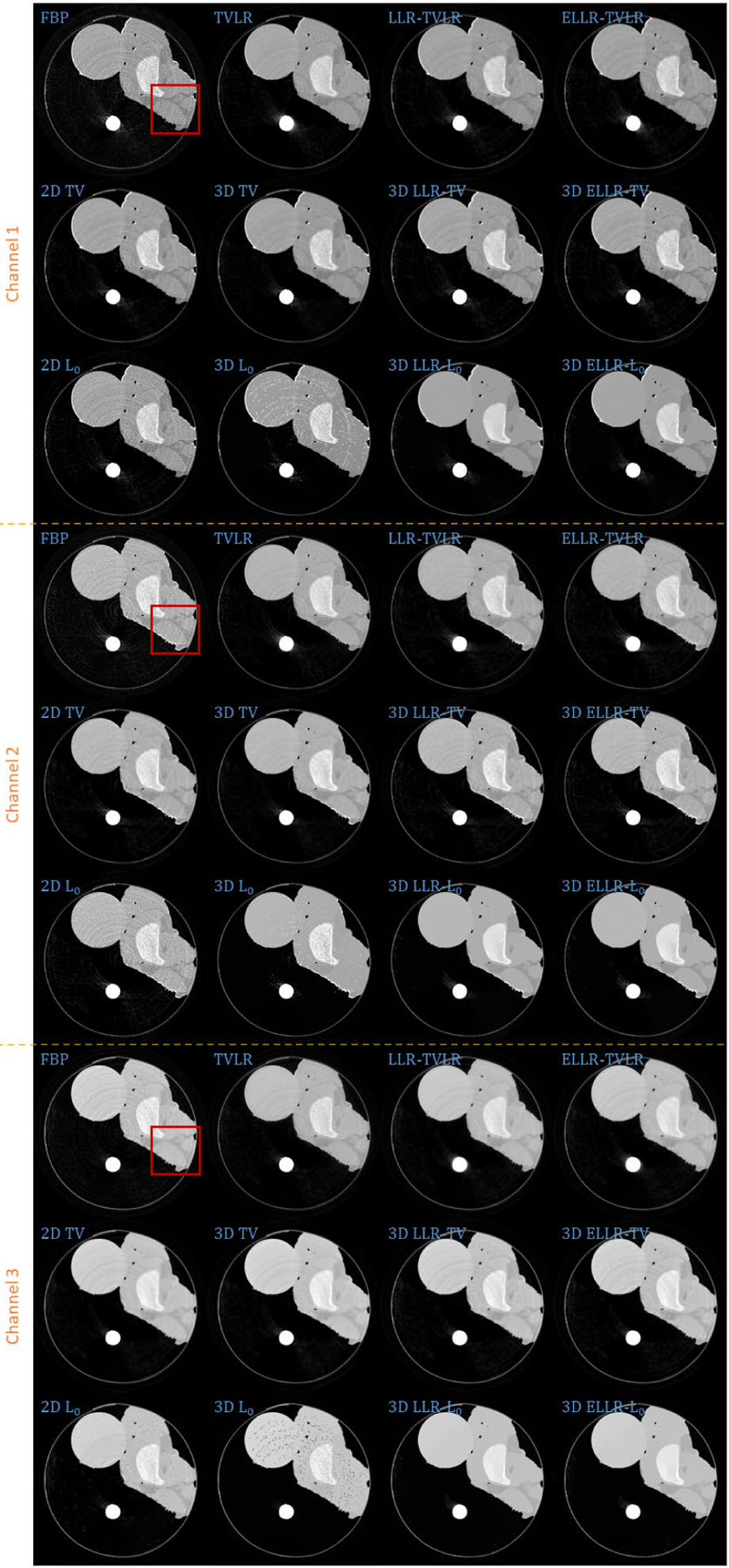
Reconstructed channel images of real experiment 1. The display window is [0,0.3] for channel 1, [0,0.26] for channel 2, and [0,0.22] for channel 3.

**FIGURE 3.**
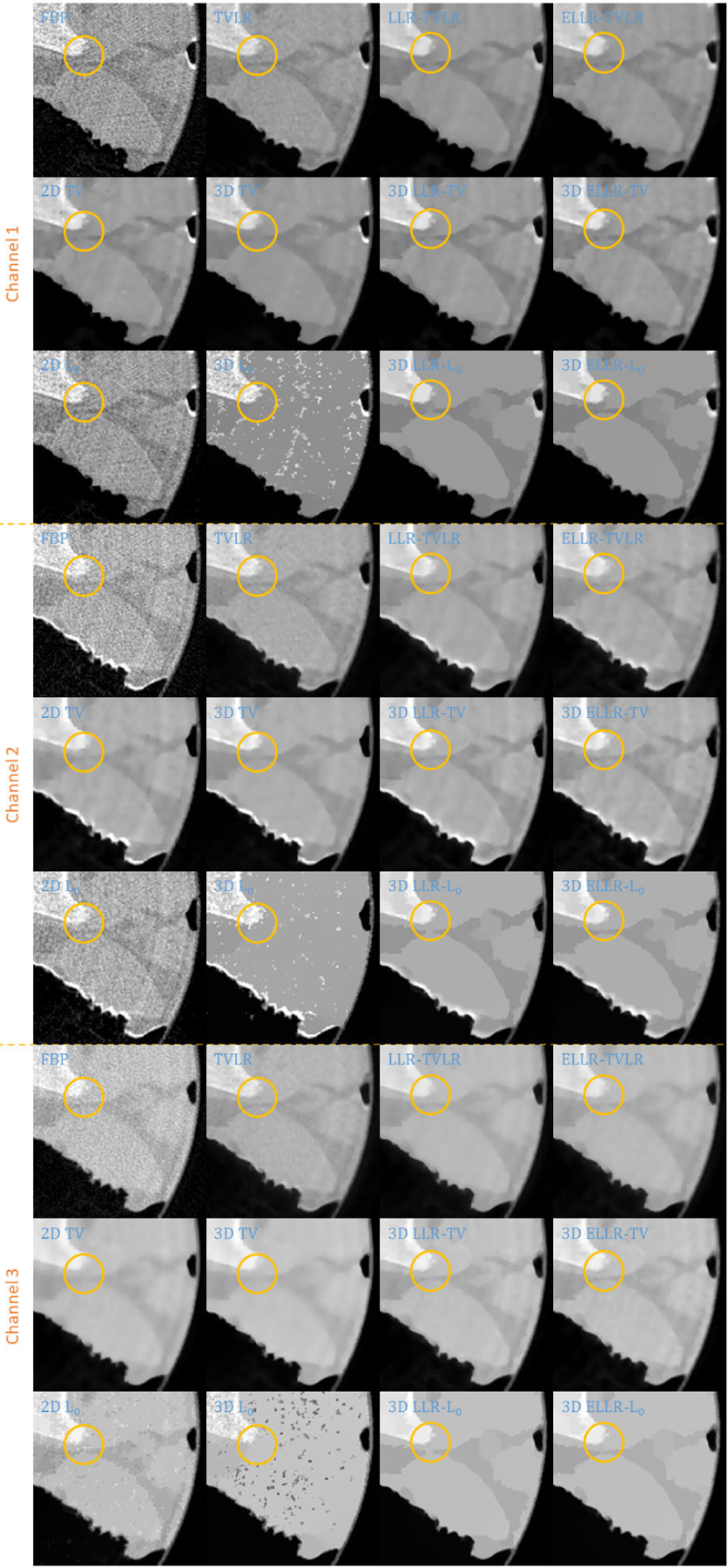
Zoomed-in patches of Fig. 2, which are marked by red boxes.

**FIGURE 4.**
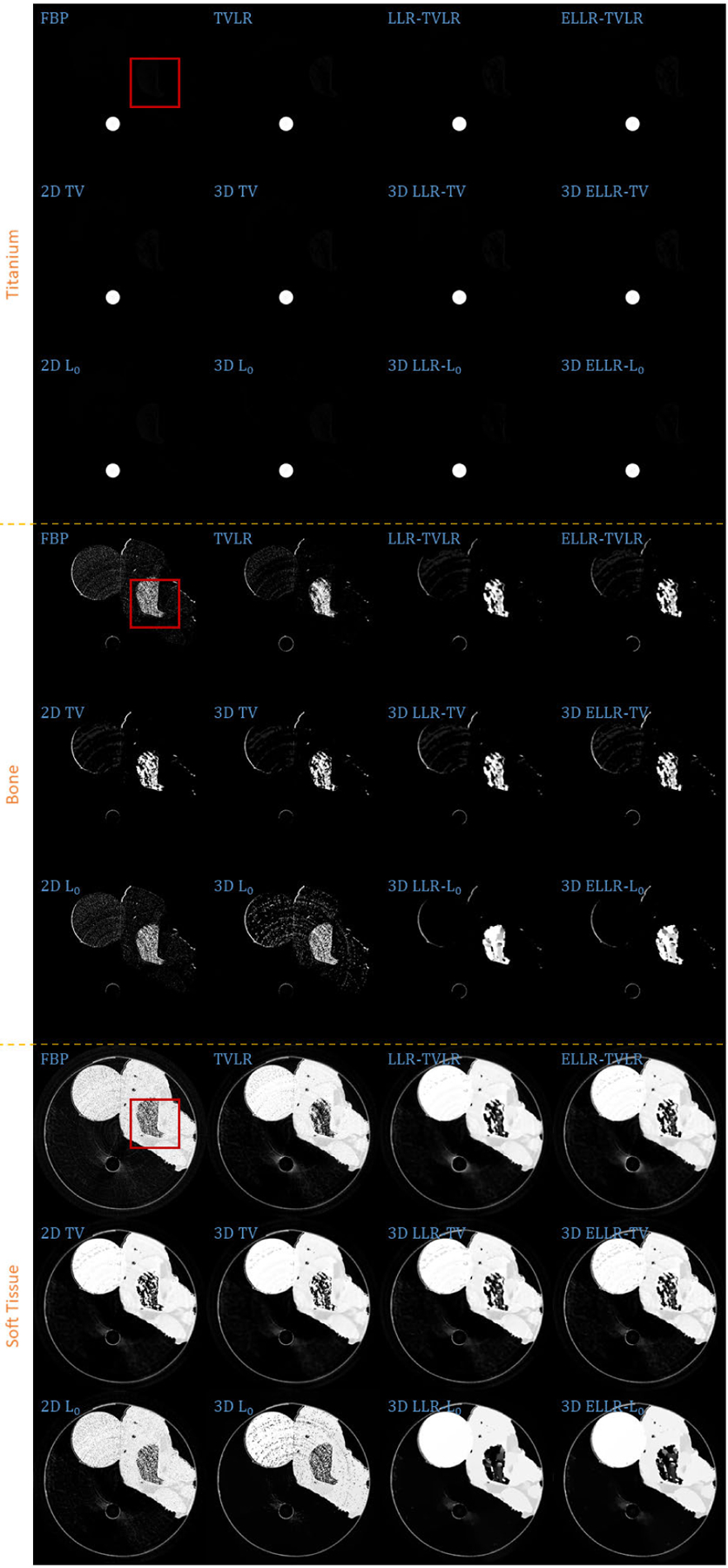
Decomposed material images of real experiment 1. The display window is [0,1] for all the results.

**FIGURE 5.**
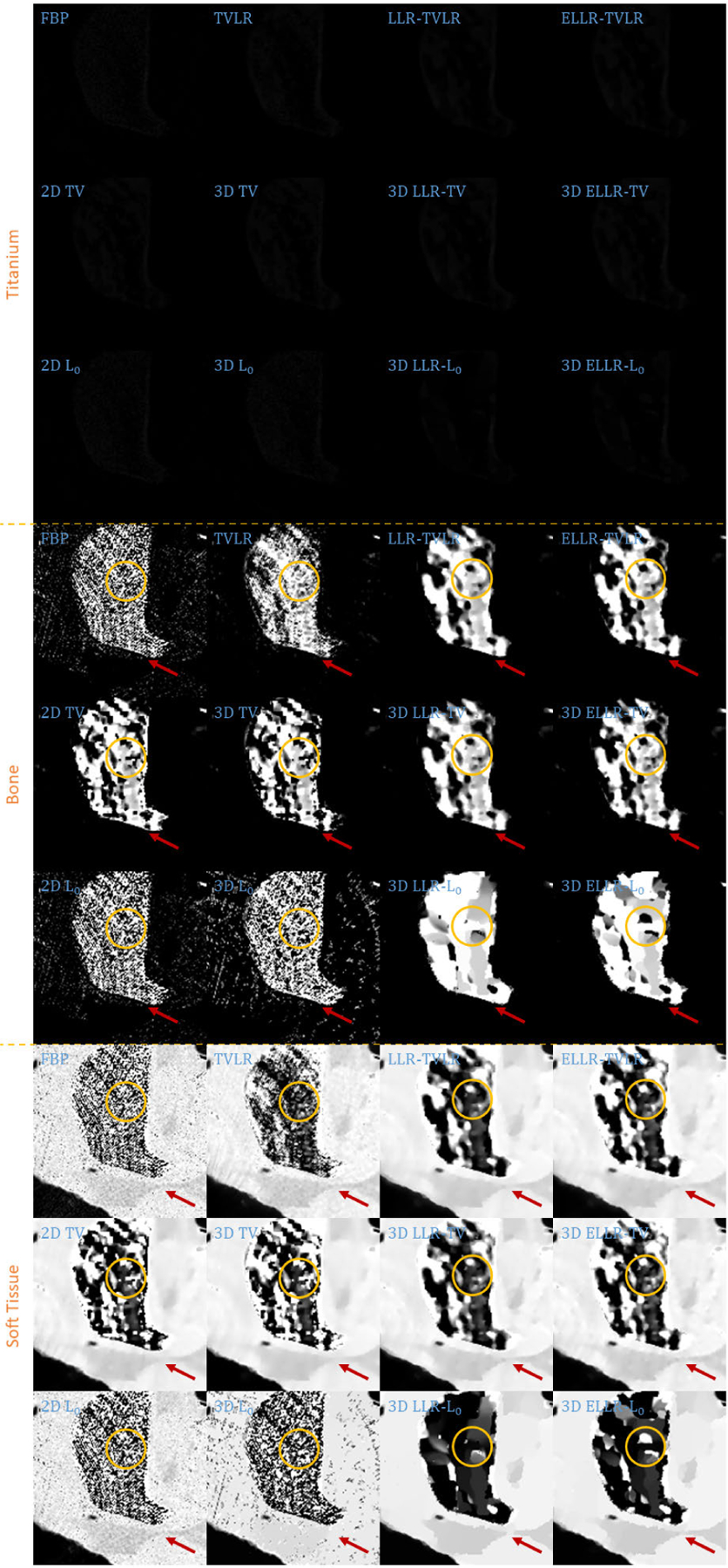
Zoomed-in patches of Fig. 4, which are marked by red boxes.

In the first real experiment, we perform a one-time scan with an equal photon ratio setting. To reduce the scattering influence, we just employ 3 energy bins with higher energies. The examined specimen, shown in Fig. 1 (upper row), is consist of chicken upper wing, titanium and solid water. The channel reconstructions and material decompositions are shown in Figs. 2 and 4. We magnify a local patch for visual comparisons in Figs. 3 and 5. To verify the decomposition accuracy, in Table 1, we quantitatively compared the decomposed solid water with mean and standard deviation measurements.

Comparing with channel images, the FBP-based results suffers from serious noise influence. The 2D based methods (2D TV and 2D *L*_0_) perform inconsistently between different channels. Some are still noisy (Fig. 3 Channel 1 2D TV and 2D *L*_0_) and some are over smoothed (Fig. 3 Channel 3 2D TV and 2D *L*_0_). The TVLR and 3D TV methods also have the inconsistent performance, such as Fig. 3 Channel 1 and 3. Because of the quantitative difference among different channel images, 3D *L*_0_ brings obvious artifacts. Both LLT- and ELLT-based methods can effectively denoise. However, the ELLT-based ones are superior in edge maintenance (comparing the patches marked by yellow circles in Fig. 3). In Figs. 4 and 5, we can find the FBP, 2D and 3D *L*_0_ results are very noisy. Although the rest methods perform well in noise suppression, the ELLT-based ones are more desirable in fine structure protection (comparing the edges marked by red arrows in Fig. 5). Another characteristic is the TV- and TVLR-class methods are good at smoothing the images because they penalize the gradient magnitude. However, *L*_0_-class works more stiff, because it penalize the gradient existence. Thus the edges are sharper than the TV- and TVLR- classes. The numerical comparison in Table 1 shows superior decomposition accuracy of the LLT-involved methods, where the mean value is very close to the ground truth and the standard deviation is very small (below 0.06).

**FIGURE 6.**
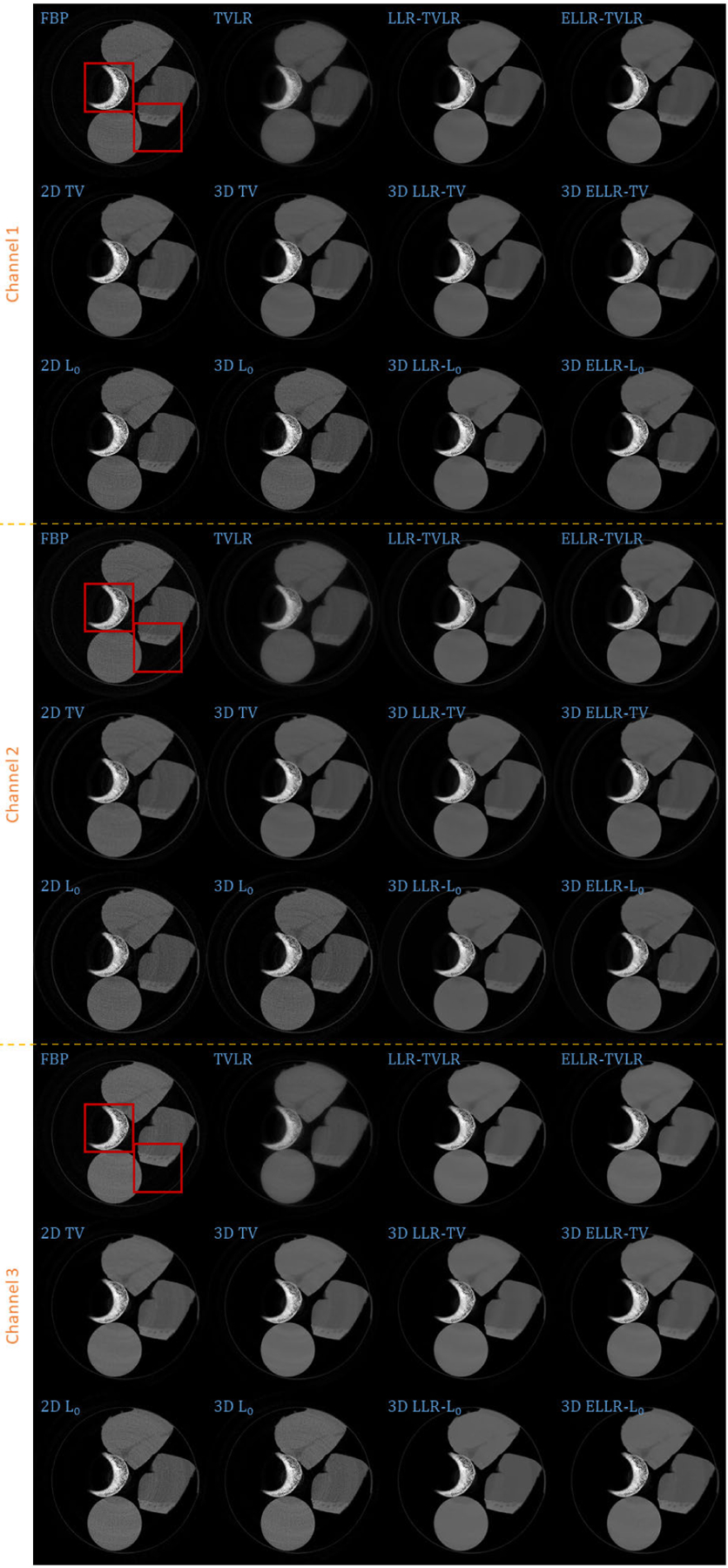
Reconstructed channel images (channel 1–3) of real experiment 2. The display window is [0,0.6] for channel 1, [0,0.6] for channel 2, and [0,0.5] for channel 3.

**FIGURE 7.**
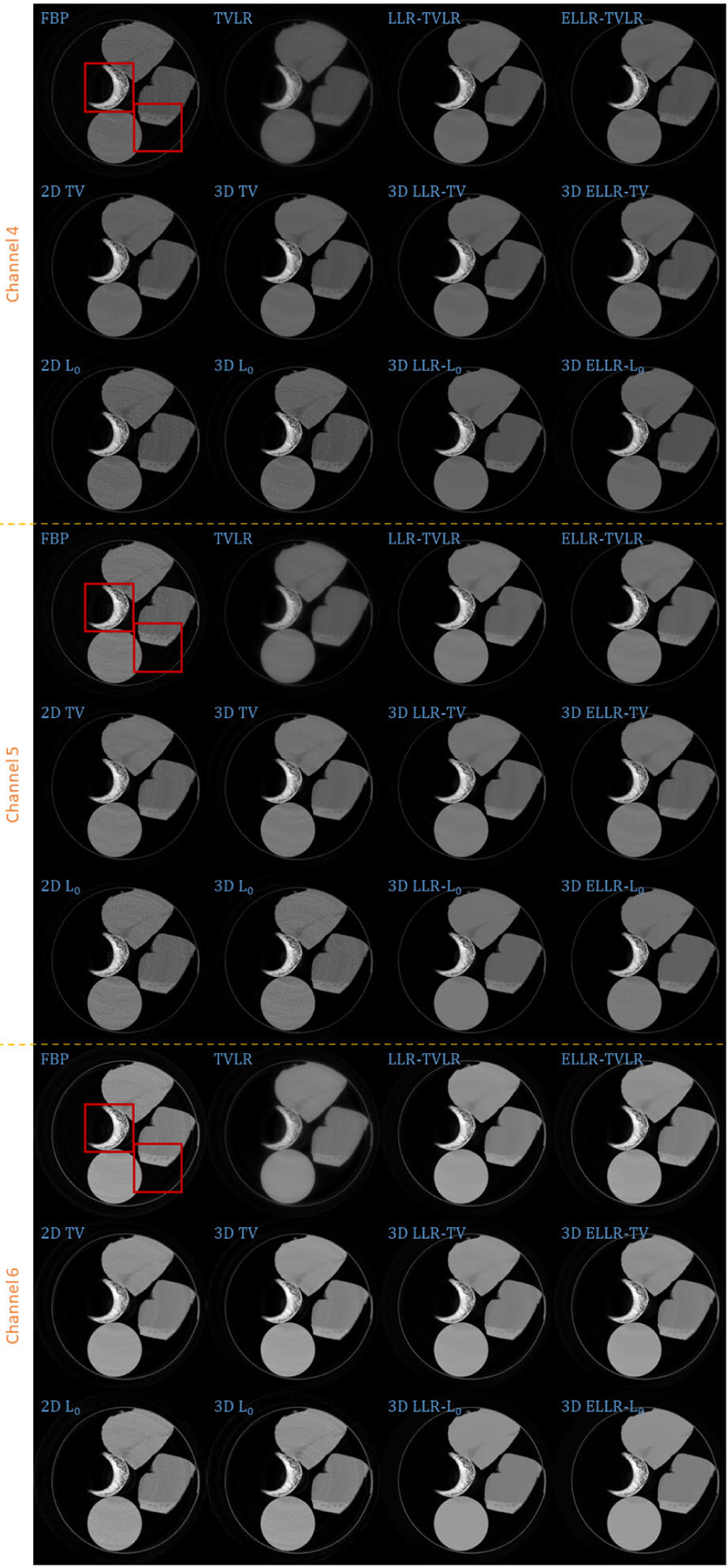
Reconstructed channel images (channel 4–6) of real experiment 2. The display window is [0,0.5] for channel 4, [0,0.4] for channel 5, and [0,0.3] for channel 6.

**FIGURE 8.**
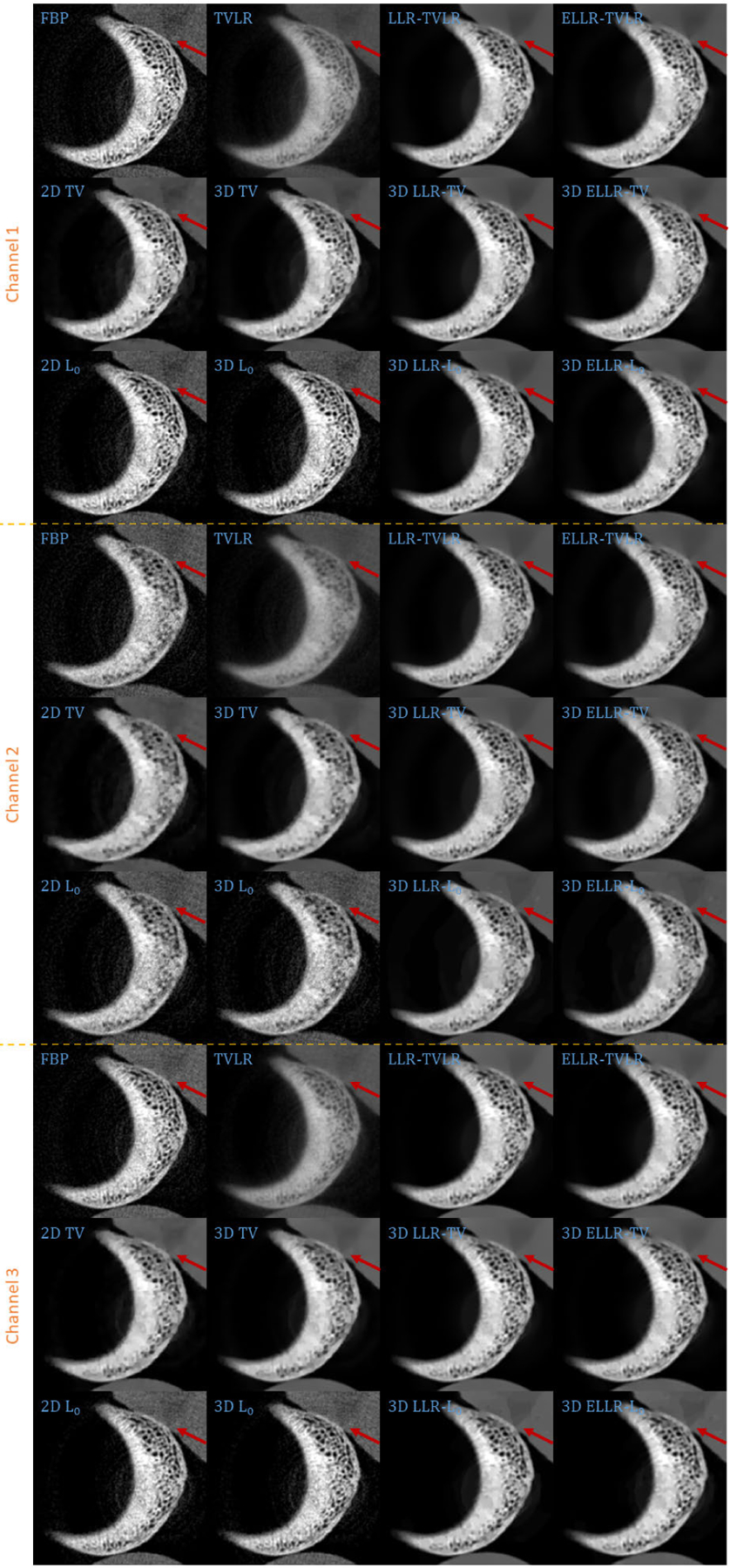
Zoomed-in patches of Fig. 6, which are marked by left red boxes.

**FIGURE 9.**
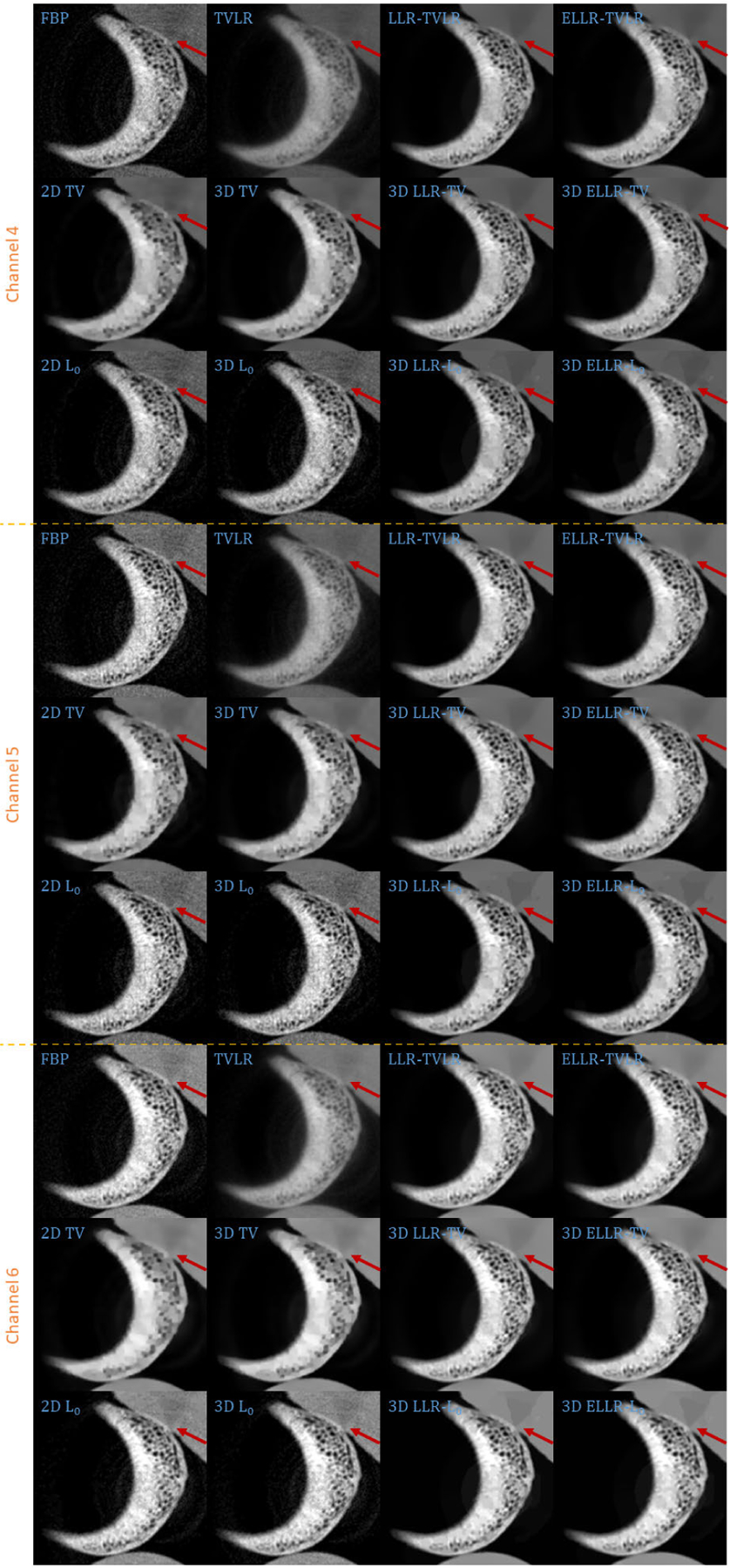
Zoomed-in patches of Fig. 7, which are marked by left red boxes.

**FIGURE 10.**
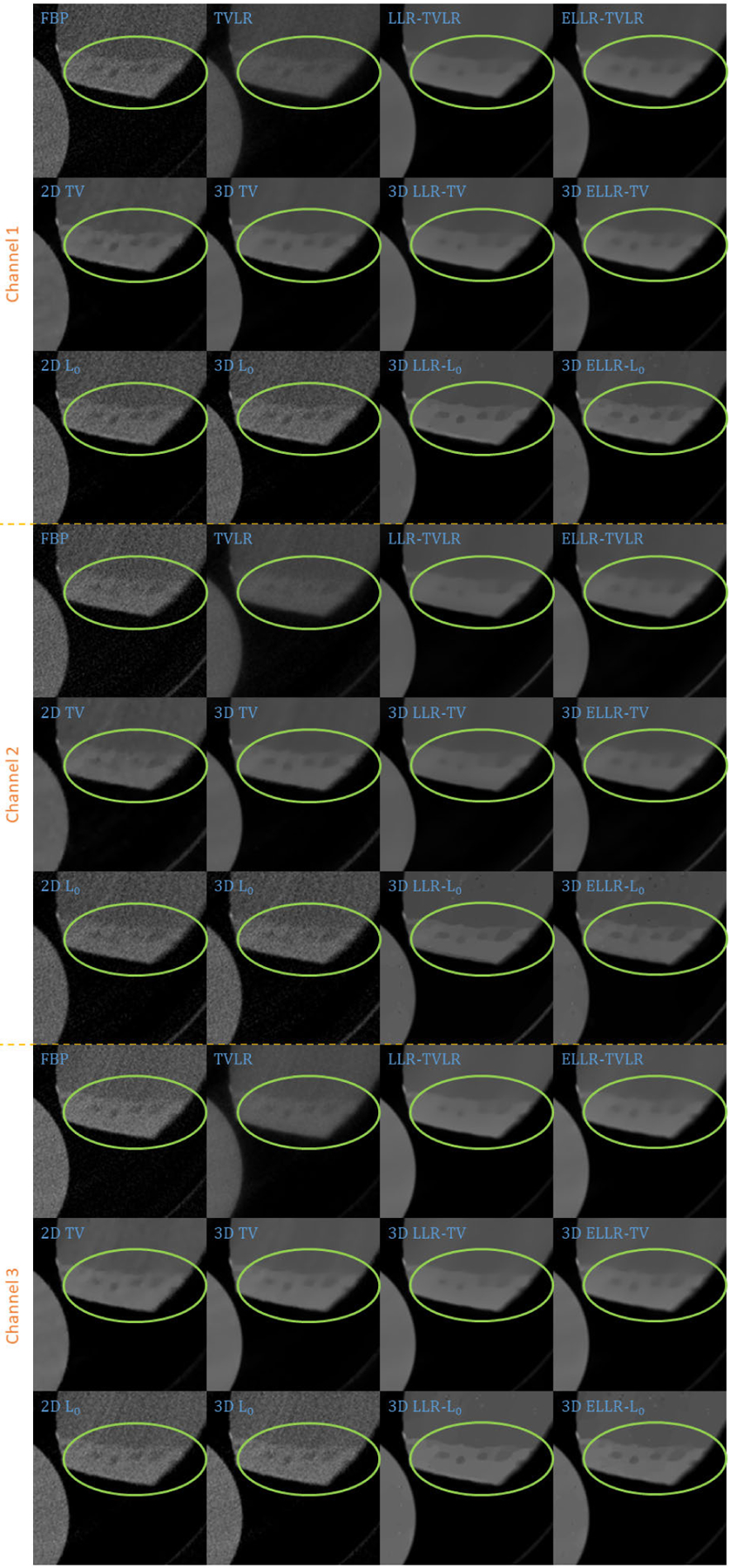
Zoomed-in patches of Fig. 6, which are marked by right red boxes.

**FIGURE 11.**
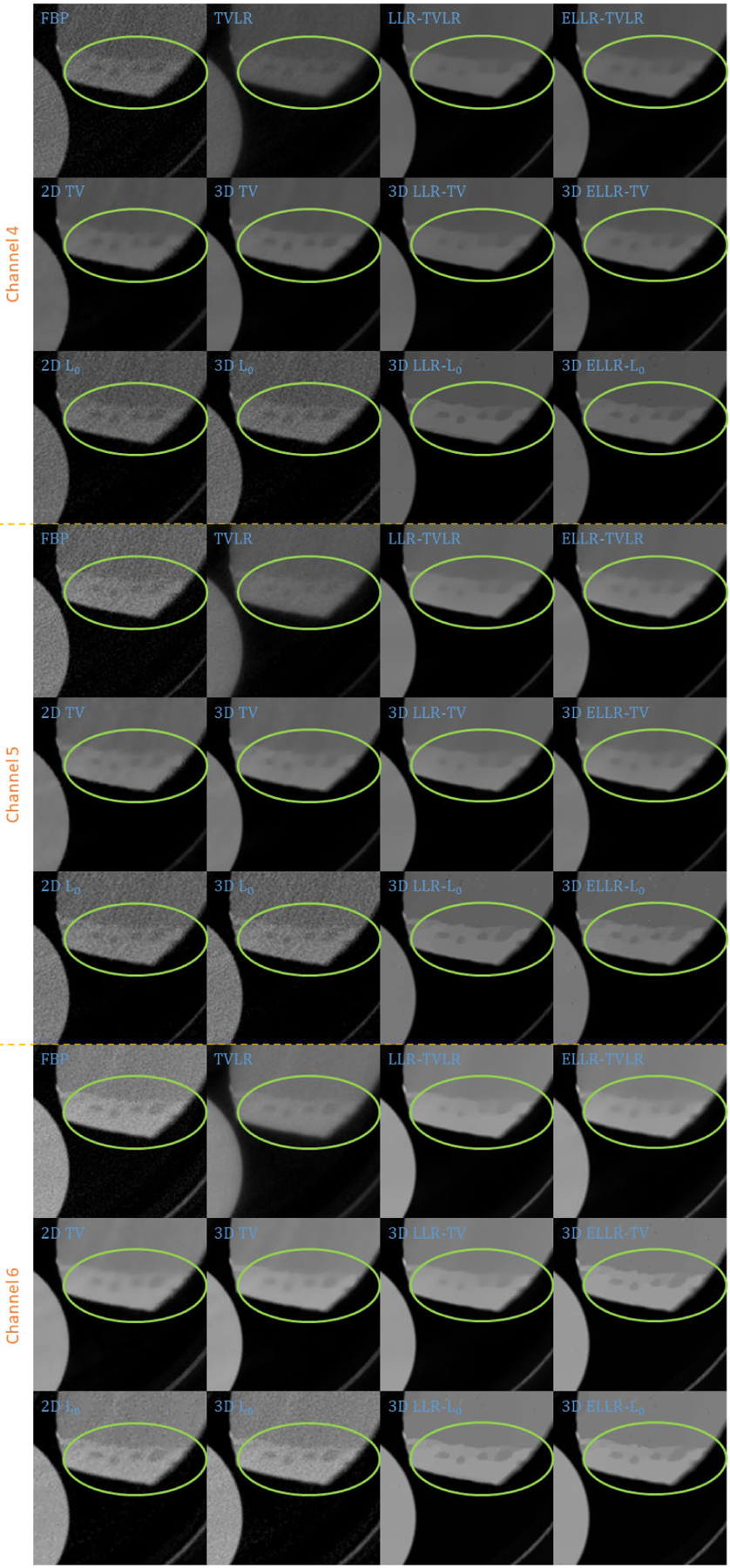
Zoomed-in patches of Fig. 7, which are marked by right red boxes.

**FIGURE 12.**
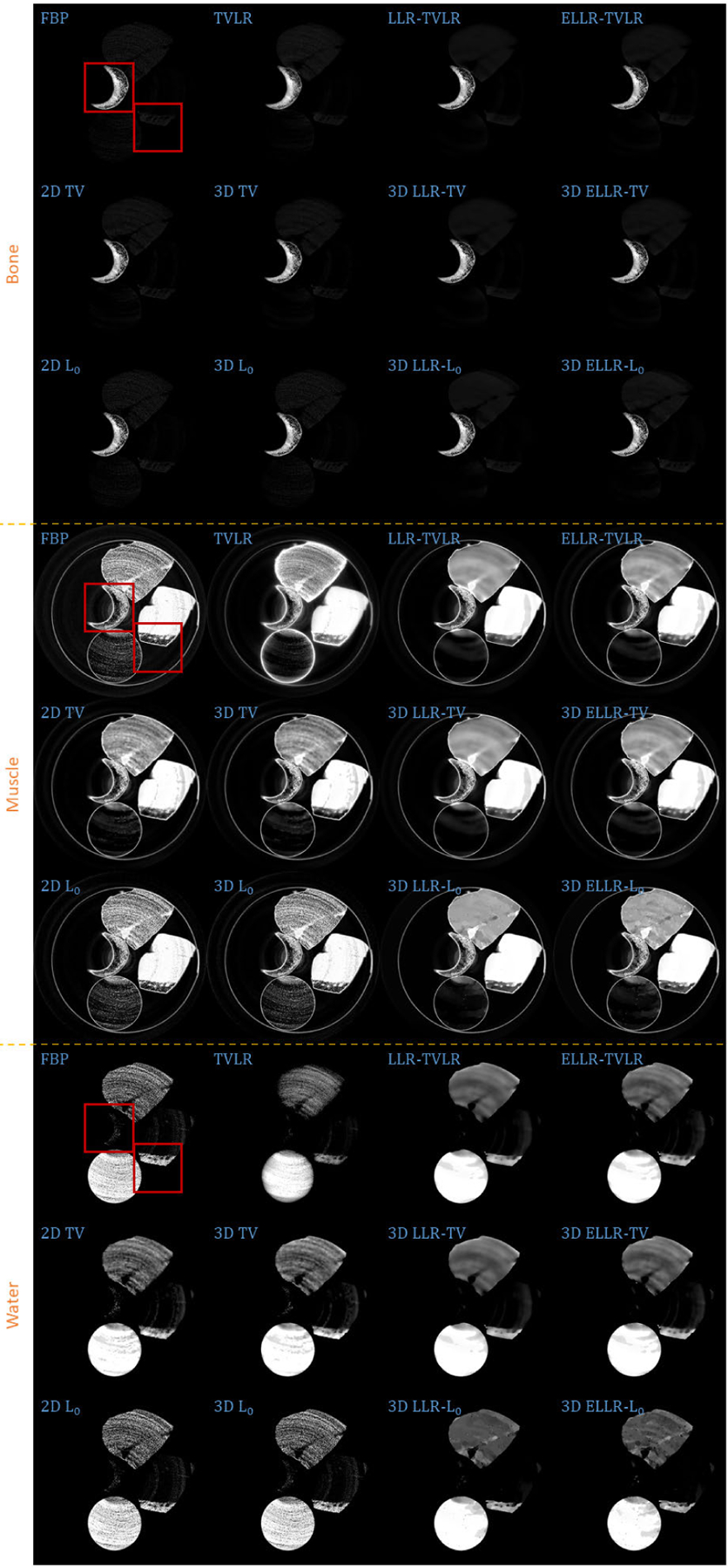
Decomposed material images of real experiment 2. The display window is [0,1] for all the results.

**FIGURE 13.**
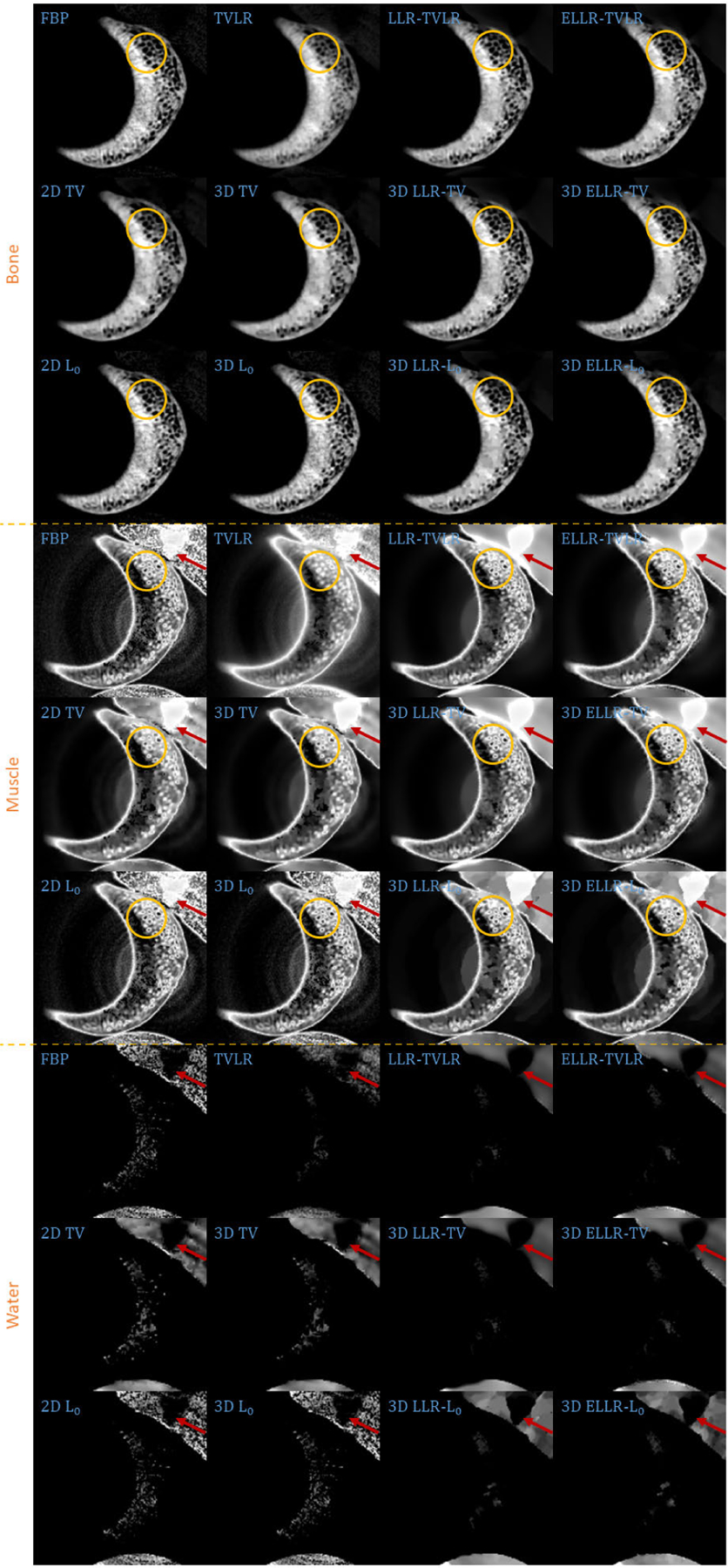
Zoomed-in patches of Fig. 12, which are marked by left red boxes.

**FIGURE 14.**
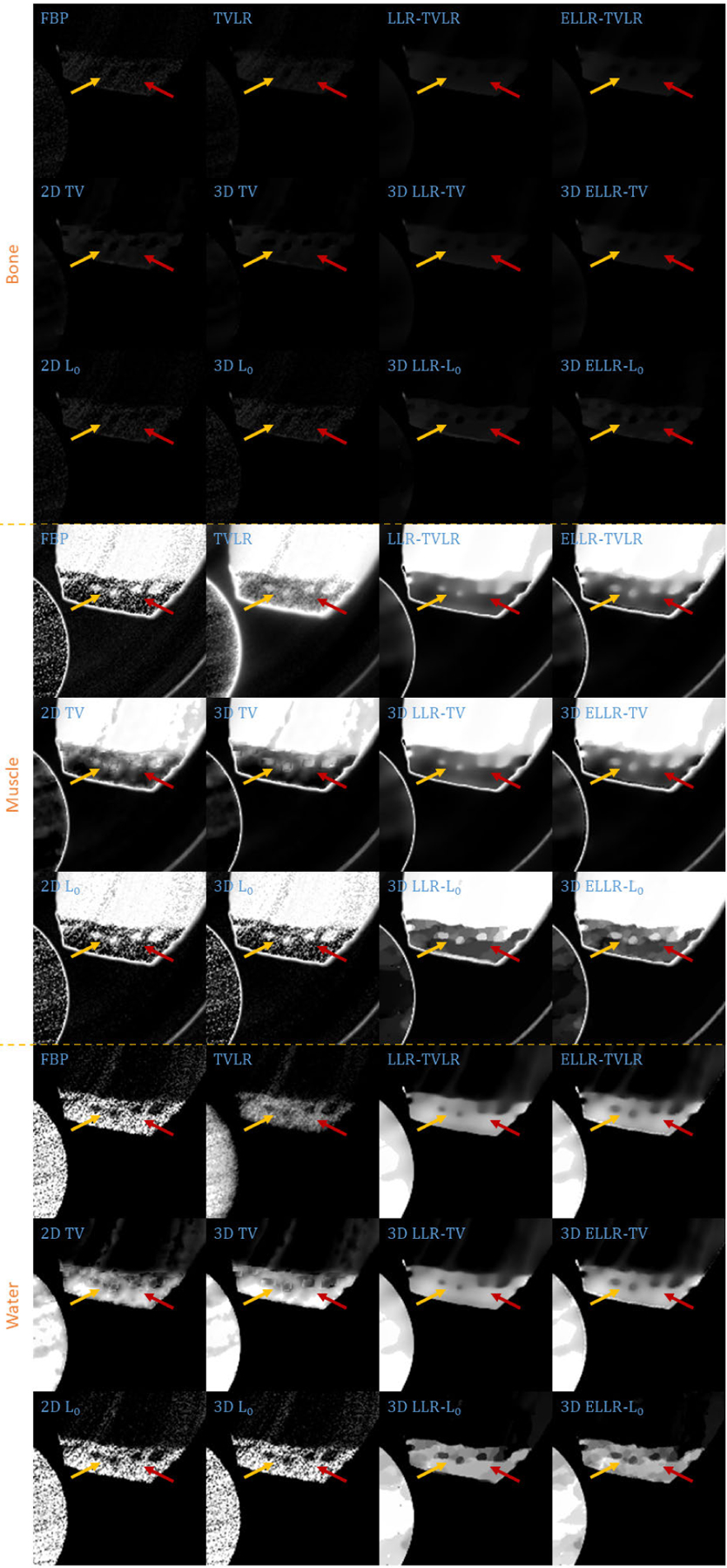
Zoomed-in patches of Fig. 12, which are marked by right red boxes.

In the second experiment, we scan twice with different energy thresholds. A 8 channel projection dataset is obtained. And for each channel, the initially emitted photon number is roughly the same. We employ 6 energy bins with higher energy to weaken the scattering influence. The examined specimen, shown in Fig. 1 (lower row), is consist of bone, muscle, fat and solid water. The channel reconstructions and zoomed-in patches are shown in Figs. 6–7 and 8-11, respectively. We choose bone, muscle and solid water as three basis materials to perform the material decomposition. The results and the magnified details are illustrated in Figs. 12 and 13-14, respectively. The numerical comparison of decomposition accuracy for solid water is summarized in Table 2.

Comparing the bone material in Figs. 8–9 and 13, the TVLR, 2D and 3D TV methods bring blurring effects to the fine bone structures. Although the FBP, 2D and 3D *L*_0_ methods well preserve the edges, they fail to effectively remove the noise in soft tissue. Both the LLT- and ELLT-based methods work well for the dual tasks and perform consistent among all the channel images. However, the ELLT-based methods are superior in fine structure preservation (see the yellow and red arrows in Fig. 14). Comparing the numerical results in Table 2, the LLT-involved methods consistently achieve high mean value (larger than 0.98) and low standard deviation (smaller than 0.04). For 2D and 3D *L*_0_ methods, the decomposed solid water had obvious bias with the ground truth, and even the standard variation is greater than 0.2. However, by introducing LLT, both of the evaluation metrics are dramatically improved, where the mean value is enhanced to 0.99 from 0.82 and the standard deviation drops to 0.04 from 0.20.

## V. CONCLUSION

In this work, we mainly investigate a method to effectively establish a sparsity feature in the spectral domain, and correspondingly develop the potential applications, such as 3D gradient *L*_1_- and *L*_0_-norm minimization and modified TVLR method for spectral CT reconstruction. Comparing with the previous work, we refine the sparsity construction method to improve the edge preservation accuracy, propose a general optimization framework, and develop three specific minimization models. Real experiments are performed, and the results confirm the effectiveness and superiority of our proposed approaches for both image quality and decomposition accuracy.

## Figures and Tables

**TABLE 1.** Quantitative comparison of decomposition accuracy for solid water in real experiment 1 (Soft Tissue Group of Fig. 4).

Mean ± Std.Dev.	FBP 0.8950 ± 0.1620	TVLR 0.9671 ± 0.0724
Mean ± Std.Dev.	LLR-TVLR 0.9891 ± 0.0421	ELLR-TVLR 0.9869 ± 0.0437
Mean ± Std.Dev.	2D TV 0.9830 ± 0.0636	3D TV 0.9863 ± 0.0604
Mean ± Std.Dev.	3D LLR-TV 0.9841 ± 0.0551	3D ELLR-TV 0.9817 ± 0.0574
Mean ± Std.Dev.	2D *L*_0_ 0.9016 ± 0.1543	2D *L*_0_ 0.9326 ± 0.2078
Mean ± Std.Dev.	3D LLR-*L*_0_ 0.9944 ± 0.0472	3D ELLR-*L*_0_ 0.9944 ± 0.0492

**TABLE 2.** Quantitative comparison of decomposition accuracy for solid water in real experiment 2 (Water Group of Fig. 12).

Mean ± Std.Dev.	FBP 0.8138 ± 0.2190	TVLR 0.9702 ± 0.0567
Mean ± Std.Dev.	LLR-TVLR 0.9879 ± 0.0183	ELLR-TVLR 0.9834 ± 0.0277
Mean ± Std.Dev.	2D TV 0.9661 ± 0.0508	3D TV 0.9686 ± 0.0470
Mean ± Std.Dev.	3D LLR-TV 0.9877 ± 0.0186	3D ELLR-TV 0.9842 ± 0.0262
Mean ± Std.Dev.	2D *L*_0_ 0.8246 ± 0.2048	2D *L*_0_ 0.8247 ± 0.2076
Mean ± Std.Dev.	3D LLR-*L*_0_ 0.9936 ± 0.0281	3D ELLR-*L*_0_ 0.9905 ± 0.0354
